# Neuroprotection of a Novel Cyclopeptide C*HSDGIC* from the Cyclization of PACAP (1–5) in Cellular and Rodent Models of Retinal Ganglion Cell Apoptosis

**DOI:** 10.1371/journal.pone.0108090

**Published:** 2014-10-06

**Authors:** Huanhuan Cheng, Yong Ding, Rongjie Yu, Jiansu Chen, Chunyun Wu

**Affiliations:** 1 Department of Ophthalmology, The Third Affiliated Hospital of Sun Yat-sen University, Guangzhou, China; 2 Department of Ophthalmology, The First Affiliated Hospital of Jinan University, Guangzhou, China; 3 Bio-engineering Institute of Jinan University, Jinan University, Guangzhou, China; 4 Department of Ophthalmology, Medical College, Jinan University, Guangzhou, China; 5 Department of Parasitology, Medical College, Jinan University, Guangzhou, China; Universidade Federal do Rio de Janeiro, Brazil

## Abstract

**Purpose:**

To investigate the protective effects of a novel cyclopeptide C*HSDGIC* (CHC) from the cyclization of Pituitary adenylate cyclase-activating polypeptide (PACAP) (1–5) in cellular and rodent models of retinal ganglion cell apoptosis.

**Methodology/Principal Findings:**

Double-labeling immunohistochemistry was used to detect the expression of Thy-1 and PACAP receptor type 1 in a retinal ganglion cell line RGC-5. The apoptosis of RGC-5 cells was induced by 0.02 J/cm^2^ Ultraviolet B irradiation. MTT assay, flow cytometry, fluorescence microscopy were used to investigate the viability, the level of reactive oxygen species (ROS) and apoptosis of RGC-5 cells respectively. CHC attenuated apoptotic cell death induced by Ultraviolet B irradiation and inhibited the excessive generation of ROS. Moreover, CHC treatment resulted in decreased expression of Bax and concomitant increase of Bcl-2, as was revealed by western-blot analysis. The in vivo apoptosis of retinal ganglion cells was induced by injecting 50 mM N-methyl-D-aspartate (NMDA) (100 nmol in a 2 µL saline solution) intravitreally, and different dosages of CHC were administered. At day 7, rats in CHC+ NMDA-treated groups showed obvious aversion to light when compared to NMDA rats. Electroretinogram recordings revealed a marked decrease in the amplitudes of a-wave, b-wave, and photopic negative response due to NMDA damage. In retina receiving intravitreal NMDA and CHC co-treatment, these values were significantly increased. CHC treatment also resulted in less NMDA-induced cell loss and a decrease in the proportion of dUTP end-labeling-positive cells in ganglion cell line.

**Conclusions:**

C*HSDGIC*, a novel cyclopeptide from PACAP (1–5) attenuates apoptosis in RGC-5 cells and inhibits NMDA-induced retinal neuronal death. The beneficial effects may occur via the mitochondria pathway. PACAP derivatives like CHC may serve as a promising candidate for neuroprotection in glaucoma.

## Introduction

Glaucoma is conventionally defined as a chronic optic neuropathy characterized by the progressive loss of retinal ganglion cells (RGCs) and optic nerve fibers [1]. Given that glaucoma is essentially a neurodegenerative disorder, the development of neuroprotective therapeutic strategies that are based not only on intraocular pressure lowering is required [2]. Neuroprotectants such as neurotrophic factors represent an important candidate treatment for glaucoma neuropathy.

Pituitary adenylate cyclase activating polypeptide (PACAP) is an endogenous neuropeptide with highly potent neuroprotective and general cytoprotective effects [3]. PACAP belongs to the vasoactive intestinal peptide (VIP)/secretin/glucagon peptide superfamily and exists in two forms, PACAP27 and PACAP38, the latter being the more biologically active [4]. The receptors for PACAP can be basically divided into two main groups: PACAP receptor type 1 (PAC1), which binds PACAP with higher affinity than VIP, and VPAC receptors (VPAC1 and VPAC2), which bind PACAP and VIP with similar affinities [5]. As a PACAP preferring receptor, PAC1 which mediates the most effects of PACAP as a neurotransmitter, neuro-modulator, neurotrophic factor and neuro-protector has been demonstrated as the predominant receptor type of PACAP in RGCs, amacrine cells, inner nuclear layer and Muller cells [6]. PAC1 is currently considered as a potential target for the treatment of neurodegenerative and neuropathic diseases. Via the mediation of PAC1, PACAP has well-known neuroprotective effects in retinal neuronal cultures *in vitro* and against *in vivo* retinal degenerations including excitotoxic injury induced by glutamate and kainate, ischemic injury, degeneration caused by UV-A light, optic nerve transection and streptozotocin-induced diabetic retinopathy [7].

However, the use of native PACAP, which is the endogenous ligand of PAC1, as an efficient neuroprotective agent is limited by PACAP's rapid degradation. PACAP can be easily hydrolyzed by the ubiquitous enzyme dipeptidyl-peptidase IV (DPP IV) to form, PACAP (3–38) or PACAP (5–38), an antagonist of PAC1 in most cases [8]. The degradation by DPP IV in the blood circulation also results in the poor metabolic stability with short half-life between 2 and 10 min while PACAP is injected into mice or human [9]. N-terminal truncation of PACAP by removal of the first five amino acids results in a potent PAC1 antagonist that retains the ability to bind PACAP binding sites, does not stimulate adenylate cyclase and inhibits the ability of PACAP to stimulate adenylate cyclase. The N-terminal domain of PACAP (His1–Ser2–Asp3–Gly4) is the key structure to activate PAC1 [10]. A novel cyclopeptide C*HSDGIC* (CHC) synthesized from the cyclization of the N-terminus of PACAP with disulfide has been revealed as a potent activator of PAC1 in our previous studies [11].As a synthetic derivative of PACAP, CHC was synthesized to overcome the poor stability of PACAP.

As is originally reported, RGC-5 is a clonal rat retinal cell line that displays RGC characteristics based on the expression of specific markers such as Thy-1, Brn-3c, Neuritin, N-methyl-D-aspartate (NMDA) and GABAb receptors, sensitivity to glutamate excitotoxicity and neurotrophin withdrawal [12]. RGC-5 cells constitute a widely used model for studying physiological and pathophysiological processes in retinal cells [13]; therefore we explored the effects of CHC on this cell line. To further evaluate the protective effects of CHC, retinal damage was induced by the intravitreal injection of NMDA into rat eyes. The cells in the ganglion cell layer (GCL) were exquisitely sensitive to both glutamate and its analog NMDA [14]. NMDA-induced reactive oxygen species (ROS)-production are well recognized to be mediators of ischemic retinal damage, and activation of NMDA receptors reportedly generated free radicals and reduced antioxidant ability in the rat hippocampus [15].Therefore, in the present study we investigated the protective effects of CHC against Ultraviolet B (UVB)-induced apoptosis in RGC-5 cells *in vitro* and NMDA-induced retinal degeneration *in vivo*.

## Materials and Methods

### Cell culture

RGC-5 cells (a generous gift from Zhongshan Ophthalmic Center, Sun Yat-sen University) was previously provided by Dr. Neeraj Agarwal (Department of Cell Biology and Genetics, UNT Health Science Center, Fort Worth, TX) [16]. The RGC-5 cells were cultured in Dulbecco's modified Eagle medium (DMEM, HyClone, USA) containing 1 g/L glucose, 10% fetal bovine serum (GibcoBRL, Invitrogen, Darmstadt), 100 U/mL penicillin, and 100 µg/mL streptomycin (HyClone, USA) under a humidified atmosphere of 95% air and 5% CO_2_ at 37°C.

### UVB irradiation

Exposure was produced by filtering banks of UVB fluorescence tubes (Spectronics, EB-260C, USA) at the wavelength of 312 nm. Exposure time was calculated with the formula: Hλ = t · Eλ, where Hλ is the energy level indicated as exposure per unit area (J/cm^2^), t is the exposure time (second), and Eλ is the irradiance (W/cm^2^).

### Immunocytochemistry staining for Thy-1 and PAC1

The cultured cells were fixed in 4% paraformaldehyde for 30 min. Following 3 washes with PBS, cells were permeabilized in 0.2% Triton X-100 for 30 min, blocked for 1 h in 10% FBS and 1% BSA in PBS, and then incubated overnight at 4°C with primary antibodies against PAC1 (1∶200, sc-15964, Santa Cruz, USA) and Thy-1 (1∶200, sc-19614, Santa Cruz, USA). For control experiments, primary antibodies were omitted. The next day, cells were washed 3 times with PBS and incubated with secondary FITC-conjugated donkey anti-goat IgG or DyLight 594-conjugated goat anti-mouse IgG (1∶200, Santa Cruz, USA) for 1 h, and then counterstained with DAPI in 1% BSA in PBS at room temperature. Images were obtained with a fluorescence Olympus BX51 microscope (Olympus, Tokyo, Japan).

### MTT assay

Cells were seeded in 96-well plates at a density of 5.0×10^3^ cells per well until 70% confluent and then incubated with different concentrations of CHC or PACAP for 1 h. To minimize the absorption of phenol red, the medium above the cells was removed during UVB irradiation at the energy level of 0.02 J/cm^2^. After incubation for another 24 h, cells were harvested and analyzed immediately for viability using the 3-(4, 5-dimethylthiazol-2-yl)-2, 5-diphenyltetrazolium bromide (MTT, Sigma, USA) assay. MTT was added at a final concentration of 0.5 mg/ml for 4 h at 37°C. Then the medium was removed and reduced MTT (blue formazan product) was solubilized by adding 100 µl of DMSO to each well. After agitation of the plates for 15 min, the optical density of the solubilized formazan in each well was measured on a spectrophotometer at a 490 nm test wavelength and a 630 nm reference wavelength.

### Propidium Iodide (PI) and Hoechst 33342 Double Staining

Cells were stained with 10 µg/ml Hoechst 33342 and 10 µg/ml PI (Sigma, USA) for 30 min at 37°C. After being washed twice with serum free media, cells were imaged using fluorescence microscopy. PI positive cells were counted using a cell counter under a fluorescence microscope at 100 times magnification. The total number of PI positive cells in four different representative fields per well were quantified for each treatment group. Morphological changes of post UVB-radiated RGC-5 cells were also observed using bright-field phase contrast microscopy.

### Assessment of ROS

Quantification of intracellular ROS accumulation was performed by flow cytometry using the fluorescent probe 2′,7′-dichlorofluorescein diacetate (DCFH-DA, KeyGEN, China). RGC-5 cells were subjected to the appropriate treatment and then incubated for 15 min in the dark at 37°C with DCFH-DA solution at a final concentration of 10 µM. After incubation, the cells were washed with PBS and analyzed within 30 min using a flow cytometry (BD FACSAria) equipped with an air-cooled argon laser tuned to 488 nm. To confirm intracellular localization of the fluorescent probe, RGC-5 cells on coverslips were stained with DCFH-DA (10 µg/ml) for 30 min at 37°C in culture medium in a humidified chamber and then fixed with 4% paraformaldehyde for 20 min. After washing in PBS, cells were observed under fluorescence microscope.

### Western Blot Analysis

After pretreatment of 10 µM CHC or 100 nM PACAP and UVB exposure, RGC-5 cells were lysed with RIPA buffer containing a protease inhibitor cocktail (Bocai Biotechnology) and sonicated on ice. The sonicated material was centrifuged for 20 min at 15,000×rpm at 4°C, and then the supernatant was collected. Protein extracts (10 µg each sample as determined by BCA method) were separated on 10% SDS-PAGE gel and transferred onto a PVDF membrane (Bio-Rad). The membrane was incubated overnight at 4°C with primary antibodies that recognize: Bcl-2 (1∶1000, Santa Cruz,USA), Bax (1∶1000, Santa Cruz,USA) and β-actin (1∶3000, Cell Signaling,USA). Horseradish peroxidase-conjugated secondary antibody (1∶3000, Santa Cruz,USA) was used for 1 h at room temperature to detect the individual antigens and was enhanced by the use of ECL chemiluminescence reagent (Pierce). Protein expression levels were quantified by scanning the immunostaining band (Tanon2500) and analyzing the image with Image J 1.39 software.

### Animals

The *in vivo* experiments were carried out on Sprague–Dawley rats at the age of 5–6 weeks old weighing 150–200 g. All animal procedures were performed in strict accordance with the recommendations in the Guide for the Care and Use of Laboratory Animals of the National Institutes of Health. The protocol was approved by the Jinan University Institutional Animal Care and Use Committee (IACUC). All efforts were made to minimize the suffering and number of animals used.

### NMDA-Induced Retinal Injury Model

Rats were anesthetized by intraperitoneal injection of 10% chloral hydrate (4 ml/kg). The pupil was dilated with phenylephrine hydrochloride and tropicamide drops. The vitreous body was then injected with 50 mM NMDA (100 nmol; Sigma, St. Louis, MO, USA) in 2 µL saline solution. A 32G needle attached to a 10 µL microsyringe was used to deliver the solution 2 mm posterior to the temporal limbus to a depth of 1 mm. In the CHC-treated groups, CHC at a concentration of 10 pM to 100 nM was co-administered with NMDA in a total volume of 2 µL into the vitreous space. In the same manner, the eyes that received only an injection of saline were served as vehicle control.

### Behavioral test

The light-dark test was carried out by standard techniques. The test box (custom-made by Metronet Technology Ltd) consisted of a dark compartment (30 cm×50 cm×50 cm) and a larger lit compartment (50 cm×50 cm×50 cm). A small opening in the center of the dividing wall at floor level allowed rats to move freely between the bright and dark chambers. Each rat was maintained in the box for 30 min and the time that different groups of rats (n = 5 for each group) spent in the dark area was recorded.

### Electroretinography (ERG)

ERG was performed 7 days after the surgery. Rats (n = 6 for each group) were dark-adapted overnight and anesthetized with an intraperitoneal injection of 10% chloral hydrate (4 ml/kg). The pupil was dilated with 1% tropicamide and then the cornea anesthetized with 0.4% oxybuprocaine. Gold wire loop was placed on the cornea and two reference electrodes were inserted subcutaneously into the tissue behind earlobes. A steel needle was inserted into the subcutaneous tissue of the tail as the ground electrode.

The a-wave, b-wave, oscillatory potentials (OPs) and photopic negative responses (PhNR) were recorded using a Roland Consult electrophysiological diagnostic system (Brandenburg, Germany). Scotopic ERGs (a-wave, b-wave, OPs; 3.0 cds/m^2^, white flash) were first recorded after dark adaptation. After light adaption under a continuous blue background (25 cds/m^2^) for 5 min to suppress rod cell electrical activity, PhNR was recorded with red flashes (3.0 cds/m^2^).

### Histology

Histology was performed on the same rats as in the behavioral test to ensure the comparability of different research results. Rats were killed by pentobarbital overdose on the 7th day after NMDA or NMDA+CHC injection. The eyeballs (n = 6 per group) were fixed overnight in 10% formaldehyde and then embedded in paraffin. Three consecutive paraffin sections (thickness 4 µm) were used for the evaluation of neuronal cell death. Hematoxylin–eosin (HE) staining was performed according to the manufacturer's instruction. Six tissue blocks from six animals were prepared from each group. The number of cells/100 µm section length in the nasal and temporal sectors of the retina 375 to 625 µm from the optic nerve in the ganglion cell layer (GCL) was measured.

### TUNEL staining

TUNEL (terminal deoxynucleotidyl transferase-mediated deoxyuridine triphosphate (UTP)-biotin nick end-labeling) staining (In Situ Cell Death Detection Kit; Roche Biochemicals, Mannheim, Germany) was performed to detect the internucleosomal DNA fragmentation that is characteristic of apoptosis. TUNEL staining was performed in addition to minor modification according to the instructional manual (n = 5 per group). Control sections were prepared with buffer solution without TdT and digoxigenin-labeled dUTP. Light-microscope images were photographed, and the labeled cells were counted in the GCL at a distance between 375 and 625 µm from the optic disc. The amount of cells staining positive for TUNEL was calculated as the percentage of the total cells in 200 µm of GCL.

### Statistical analysis

Statistical comparisons were made by using an analysis of variance (ANOVA) followed by Student-Newman-Keuls or Tamhane's T2 test where appropriate. Data were presented as means ± S.E.M. A P value<0.05 was considered statistically significant.

## Results

### CHC attenuates the apoptosis of RGC-5 cells via the mitochondria pathway

**Figure 1:**
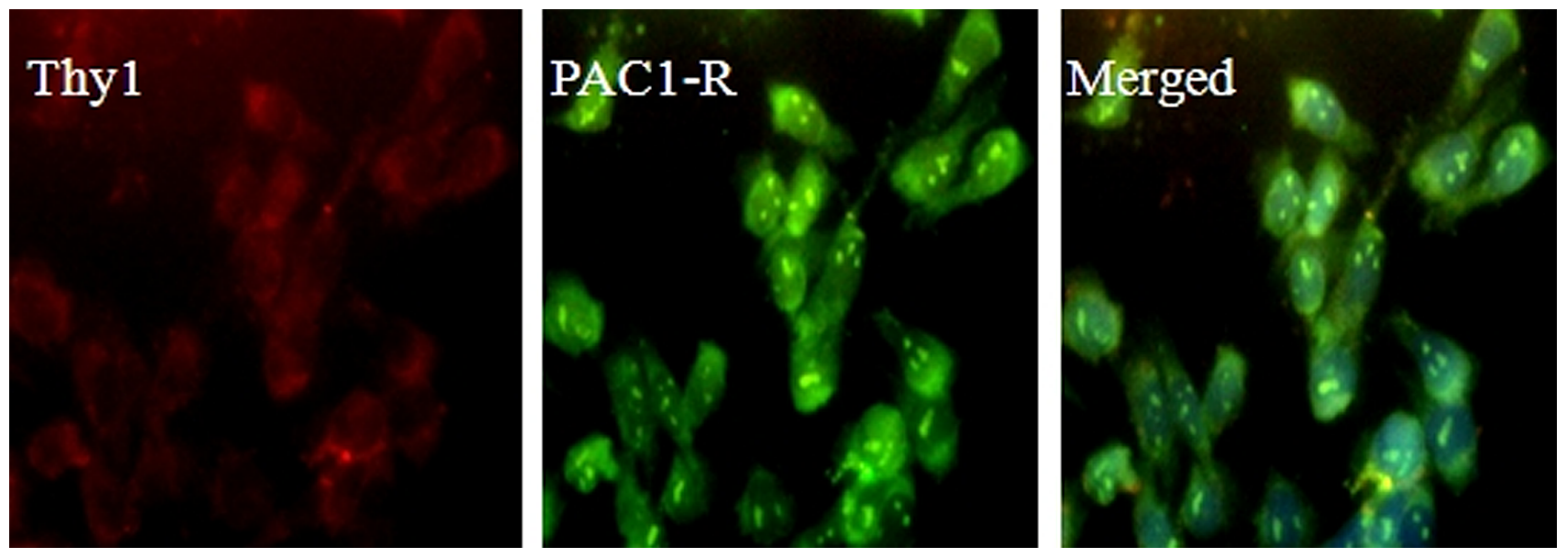
Immunolocalization studies for Thy-1(red) and PAC1 (green) with double staining. Evident labeling of the RGC marker Thy-1 was observed in RGC-5 cells. Immunostaining also showed diffuse expression of PAC1 in RGC-5 cells.

**Figure 2:**
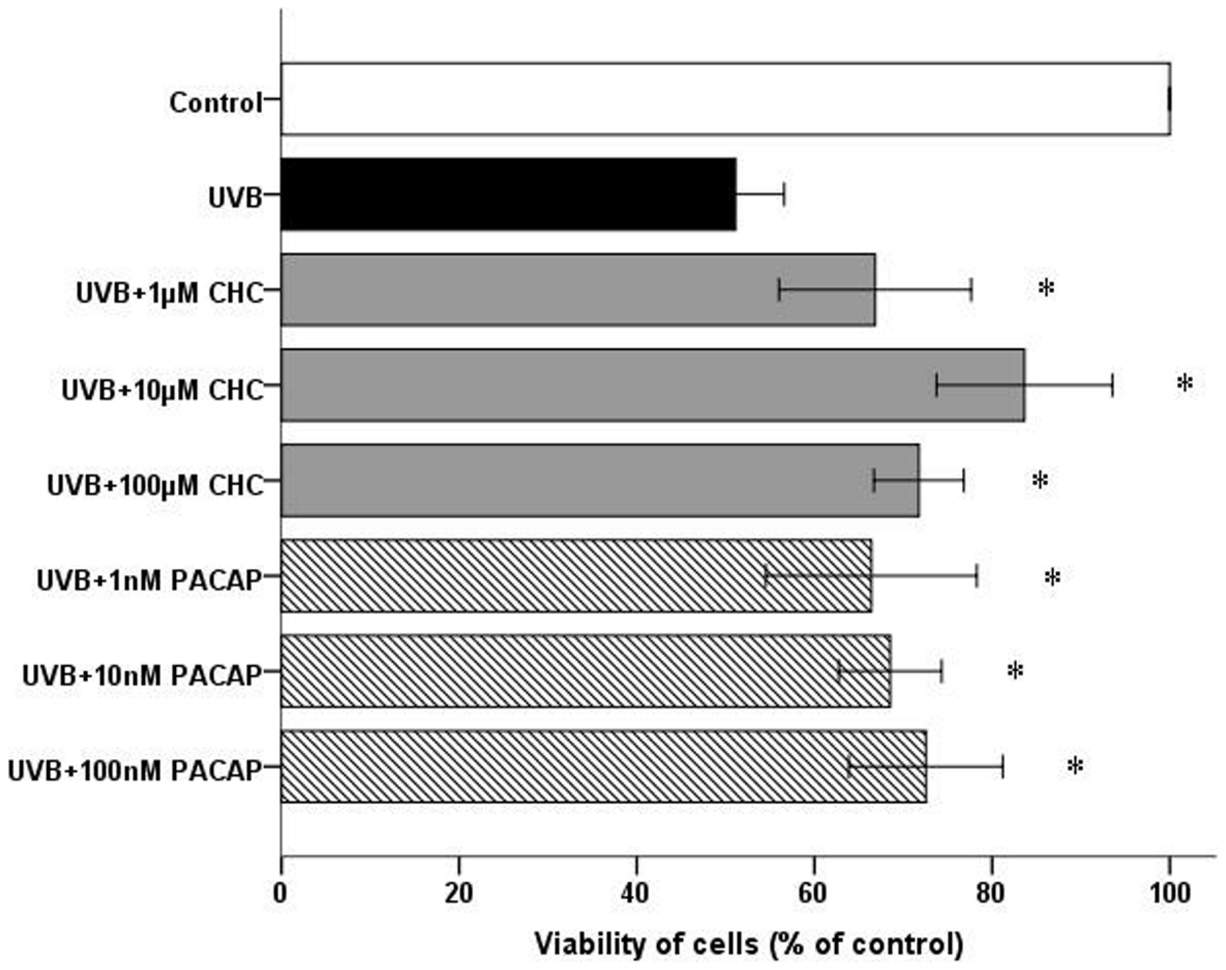
Viability of RGC-5 cells in different treatment groups at 24 h post UVB irradiation as measured by MTT assay. Co-administration of 10 µM CHC conferred the most significant protective effect compared with cells exposed to UVB only. The experiment was carried out three times independently and six replicate wells were set each time. *P<0.05, compared to UVB values.

Immunocytochemistry demonstrates strong staining for PAC1 in RGC-5 cells. Representative micrographs also showed discernible immunoreactivity for Thy-1 in all cells (Fig. 1). 0.02 J/cm^2^ was identified as the ideal UVB energy level that reliably produced an approximately 50% reduction in cell viability compared to the control. Viability of RGC-5 cells was reduced to 51.10±2.21% 24 h after 0.02 J/cm^2^ UVB irradiation. This detrimental effect was markedly attenuated by the presence of CHC or PACAP (P<0.05). 10 µM CHC afforded the greatest protection to the RGC-5 cells exposed to UVB damage, increasing viability to 80.3±2.35% (Fig. 2). Interesting discoveries were made by further distinguishing the viability of two different treatment groups. The viability of RGC-5 cells in the UVB+10 µM CHC treatment group was significantly higher than that treated with UVB and PACAP or other concentrations of CHC (P<0.05). The viability of cells with pretreatment of 1 µM CHC or 1 nM PACAP was significantly lower than that of those pretreated with CHC or PACAP at other concentrations (P<0.05), suggesting the limited protective effects CHC and PACAP at lower concentrations.

**Figure 3:**
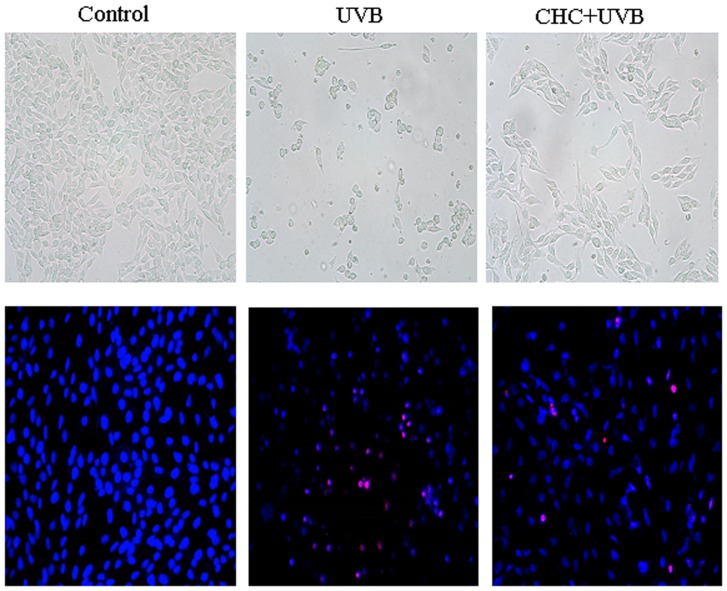
Representative light micrographs (A–C) and fluorescence micrographs of Hoechst33342 (blue)/PI (red) double staining (D–F) of normal RGC-5 cells, cells subjected to UVB irradiation and cells treated with 10 µM CHC and UVB irradiation. Normal control cells grew as an interconnected monolayer and exhibited axonal processes (A). Cells after UVB exposure became cuboidal and crenate in shape with the presence of vacuoles (B), which was markedly ameliorated with CHC pretreatment (C). Control cells showed normal nuclear morphology and are negatively stained for PI (D). UVB-irradiated cells including PI-positive cells, showed shrinkage and condensation of their nuclei (E). Treatment with 10 µM CHC reduced both nuclear shrinkage and PI-positive staining (F). Magnification is×200.

Normal control cells grew as an interconnected monolayer and exhibited axonal processes. Cells after UVB exposure became cuboidal and crenate in shape with the presence of vacuoles; while the structural morphology of RGC-5 cells was markedly maintained with CHC pretreatment, showing an elongated central axis (Fig. 3 A–C). Microscopic evaluation of apoptotic cells as a consequence of UVB exposure was determined by the Hoechst 33342 and PI double staining method. Control cells displayed normal nuclear morphology, while cells exposed to UVB irradiation showed shrinkage and condensation of their nuclei with many red staining cells which were thought to be necrotic cells. CHC (10 µM) was significantly effective in blunting the cell death caused by UVB exposure (Fig. 3 D–F). UVB damage to RGC-5 cells elevated the percentage of PI positive cells (45.3±8.4%) relative to normal control cells. This increase was reduced by the pretreatment of 10 µM CHC (19.8±8.5%).

**Figure 4:**
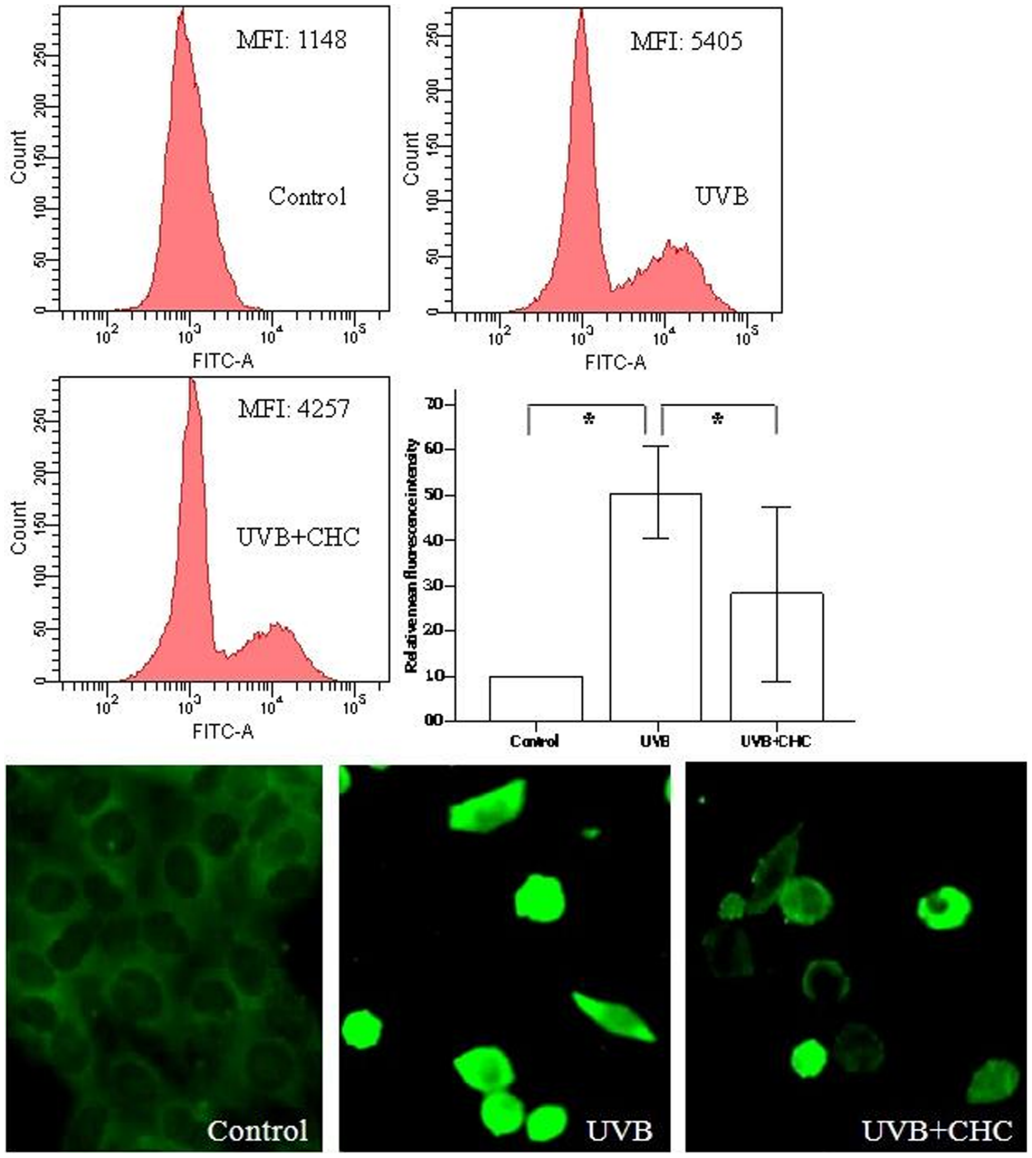
Effect of CHC on the ROS level in RGC-5 cells 24 h after UVB exposure as revealed by flowcytometry and fluorescence microscopy. RGC-5 cells exposed to UVB insult exhibited intense green fluorescent staining. 10 µM CHC clearly blunted the accumulation of ROS in UVB-exposed RGC-5 cells. Data were expressed as means±S.D. (*P<0.05).

The measurement of ROS is based on the ability of the non-polar, non-fluorescent DCFH-DA to diffuse through the cell membrane and to be deacetylated by cytosolic esterases to form the polar, non-fluorescent dichlorodihydrofluorescein (DCFH), which is trapped inside the cell and gives rise to the formation of the fluorescent derivative dichlorofluorescein (DCF) by reacting with ROS [17]. ROS production is indicated by FITC fluorescence, and the mean intensity of fluorescence (MIF) was clearly enhanced in the cells subjected to UVB irradiation and up-regulated to over four-fold of control. Such enhancement was clearly reduced by the pretreatment of 10 µM CHC. Fluorescent microscopy examination of UVB-irradiated cells showed that the cells were strongly fluorescent, with a fairly uniform distribution of the dye indicating the spread of the oxidative burst within the cells, while cells protected with CHC showed a reduction in DCF accumulation (Fig. 4b).

**Figure 5:**
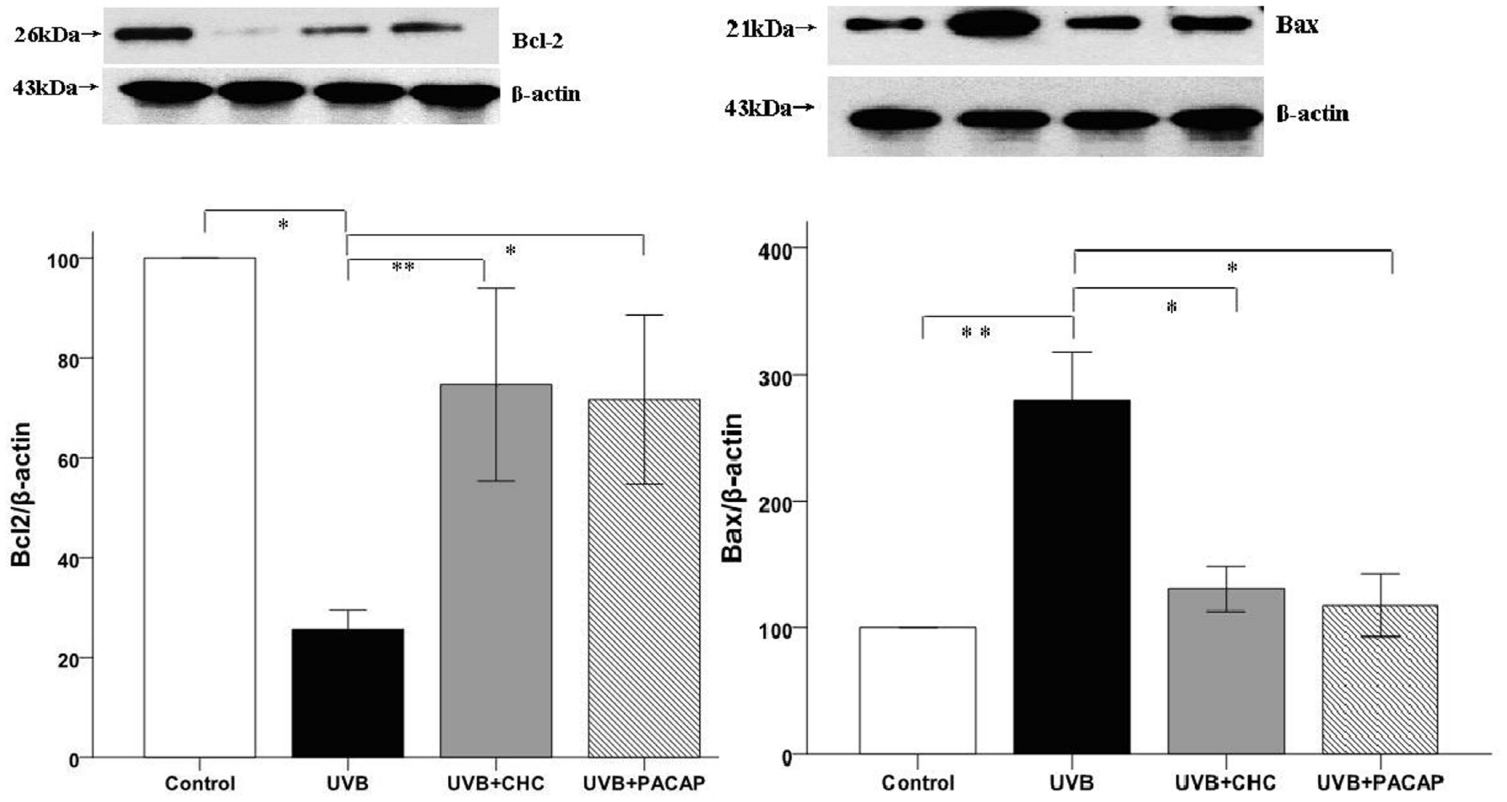
Western-blot analysis of RGC-5 cells exposed to UVB irradiation with or without the pretreatment of 10 µM CHC or 100 nM PACAP. UVB exposure caused a significant decrease in the protein level of Bcl-2 and a concomitant increase of Bax, while CHC significantly decreased the expression of apoptotic marker Bax and increased Bcl-2 expression. The results are expressed as means±S.D. (*P<0.01, **P<0.05).

The level of Bcl-2 and Bax protein relative to β-actin was investigated with western blot analysis. The result showed a marked decrease in the level of Bcl-2 with a concomitant increase in the level of Bax protein after UVB irradiation. In contrast, the effect of UVB irradiation on the expression of Bcl-2 and Bax was significantly attenuated by 10 µM CHC or 100 nM PACAP treatment. Furthermore, in terms of the expression level of these apoptosis-related markers, there was no significant difference between CHC and PACAP-treated cells, P>0.05 ((Fig. 5)).

### Improvement of visual function and histology with CHC treatment

**Figure 6:**
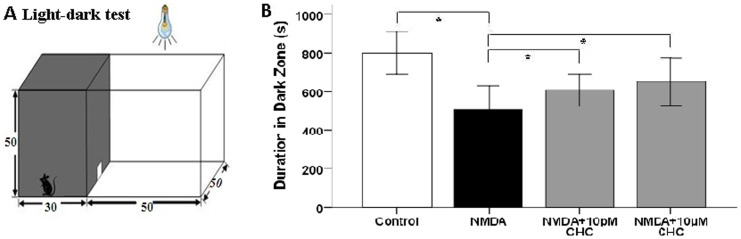
Enhancement of visual function in NMDA-injected SD rats with CHC co-application. (A) The light-dark test box consisting of a dark compartment and a larger lit compartment. (B) Time spent in the dark area by four groups of rats. The NMDA-CHC treated rats showed behavioral aversion to light. They spent significantly longer time in the dark chamber than rats injected with NMDA alone (*P<0.01).

In the light-dark test, the total residence time in the test box for each rat was 30 min (1800 s). Time spent in the dark area by NMDA-treated rats was 505.27±50.19 s, significantly lower than that of normal control rats (799.95±69.34 s).With 10 pM or 10 µM CHC co-treatment, the duration in dark zone was increased to 608.43±50.88 s and 625.00±48.97 s respectively (Fig. 6).

**Figure 7:**
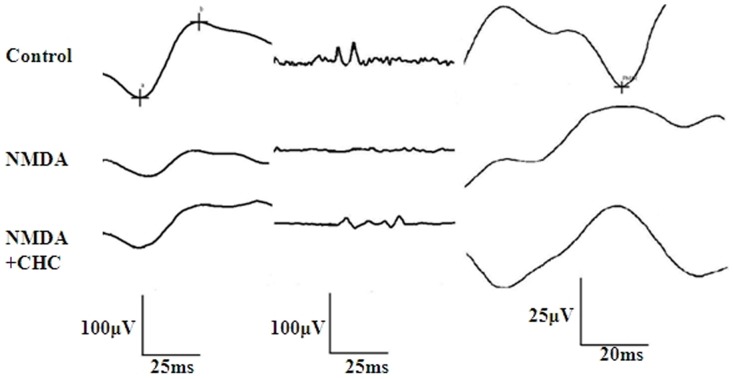
Different waves in ERG recordings of different treatment groups. Representative dark-adapted ERG components (a-wave, b-wave and OPs) and PhNR were recorded in control, NMDA and NMDA+CHC treatment groups. a-wave and b-wave are shown in the first column, while OPs and PhNR were shown in the second and third column respectively.

The amplitudes of b-wave and PhNR were reduced in NMDA-injected rats compared with age-matched controls, whereas CHC co-administration markedly increased these amplitudes (Fig. 7). Amplitudes of OPs in NMDA-treated rats after 7 days were markedly lower than normal controls (P<0.05). 10 µM CHC co-administration slightly reversed NMDA-induced Ops reduction, but the difference was not statistically significant. As for the PhNR amplitudes, in NMDA group they were smaller than the control level, while with 10 µM CHC treatment the values were significantly increased,P<0.05 (Table 1).

**Table 1:** Amplitudes of ERG components in different groups.

Groups	N	b-wave (µV)	Ops (µV)	PhNR (µV)
Control	6	123.67±16.04	146.33±15.01	36.4±6.15
NMDA	6	44.83±2.75	42.93±15.00	12.74±5.05
NMDA+CHC	6	68.7±5.75*	58.83±4.18	22.57±0.87*

*P<0.05, compared with the NMDA group.

**Figure 8:**
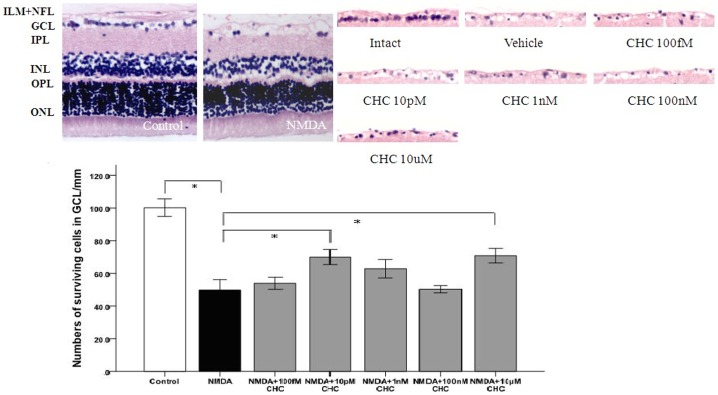
Representative photomicrograph showing HE staining of cell layers in the retina (100×). The number of cells per 100 µm in GCL decreased in NMDA-treated retinas but was increased by CHC treatment. The 10 pM and 10 µM CHC -treated groups are significantly different from the control vehicle group. *P<0.01 (ONL outer nuclear layer, OPL outer plexiform layer, INL inner nuclear layer, IPL inner plexiform layer, GCL ganglion cell layer, ILM inner limiting membrane).

In the intraocular NMDA-induced excitotoxicity model, a significant decrease was observed at day 7 in the number of cells in the GCL of vehicle-injected retina compared with the intact control (49.74±5.16/mm vs100.2±4.31/mm; p<0.01). In those groups in which CHC was injected into the vitreous body, the cell number in GCL was 53.94±3.0, 70.0±3.7, 62.9±4.54, 50.34±1.8 and 70.8±3.61/mm for CHC at concentrations of 100 fM, 10 pM, 1 nM, 100 nM and 10 µM respectively. The increase was significant in the groups treated with 10 pM or 10 µM CHC compared with the vehicle-treated group, suggesting that the neuroprotective effect of CHC on RGCs was bimodal (Fig. 8).

**Figure 9:**
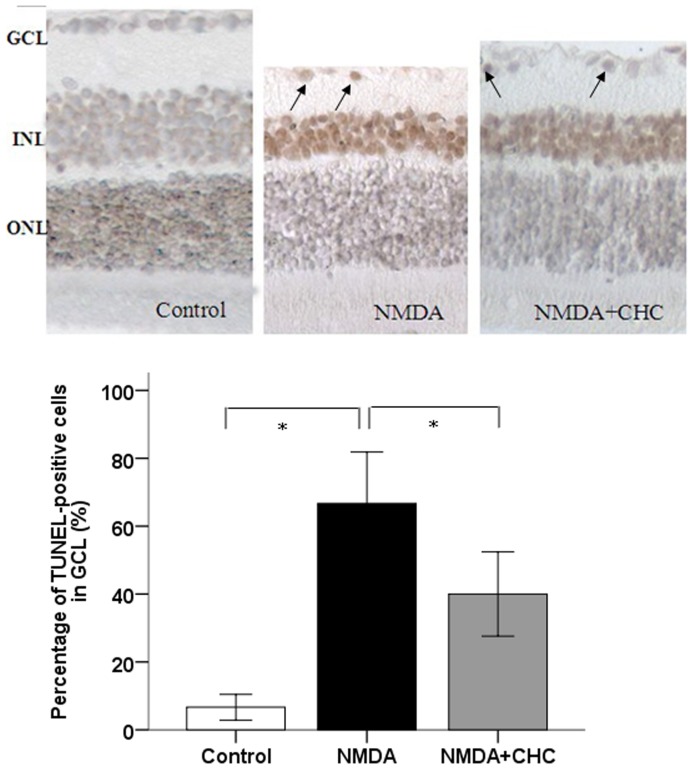
Representative photomicrogaphs illustrating the apoptosis of RGCs in response to NMDA (100×). Deep brown-stained cells indicate TUNEL-positive cells. The proportion of TUNEL-positive cells in the GCL of control SD rats, rats injected with NMDA alone or with NMDA+10 µM CHC were calculated. Data are shown as means±S.E.M. (n = 6). *P<0.05, comparing the NMDA and NMDA+CHC groups.

In the case of TUNEL-positive cells, the percentage of TUNEL-positive cells in the intact control retina was 6.67±1.53%. After NMDA injection, a significant increase was recorded in the percentage of TUNEL-positive cells (66.67±6.11%), with CHC co-treatment the value dropped to 40.0±5.0% (Fig. 9).

## Discussion

In the present study, we investigated the protective effects of a PACAP derivative in both *in vitro* and *in vivo* models of RGC apoptosis. To our knowledge, this is the first study investigating the anti-apoptotic effects and mechanism of a synthetic PACAP derivative in RGCs. These results are consistent with the large body of evidence demonstrating the retinoprotective action of PACAP in numerous models of retinal degeneration including excitotoxic retinal injury induced by NMDA in mice [18] and by monosodium glutamate in newborn rats [19]. PACAP treatment has also been shown to significantly attenuate the UVA-induced retinal damage [20], which was in accordance with our findings that PACAP and CHC inhibited UVB-induced apoptosis in RGC-5 cells.

Structural analysis has shown that the N-terminus of PACAP (HSDGI) is flexible and disordered [21], while C*HSDGIC* synthesized from cyclizing HSDGI and adding two Cys residues at both ends is more stable with a more fixed conformation. Furthermore, CHC has a small molecular weight (approximately 700 Da), which allows it to easily penetrate the blood-retina barrier. CHC is also a selective agonist of PAC1 without inducing side effects mediated through other receptors of PACAP [11].

PAC1 has gained considerable attention because it has been shown to mediate the anti-apoptotic and cytoprotective effects of PACAP [22] and accumulating studies have noticed the value of detecting PAC1 expression in retinal cells [23]. To confirm the results of western blot analysis in our previous studies [24], immunocytochemistry was performed on RGC-5 cells with Thy-1 and PAC1 antibodies. The RGC-5 cell line used in this study was positive for Thy-1, a specific marker for RGCs. Immunofluorescence also showed diffuse expression of PAC1 antigen, the putative target for PACAP analogs.

Currently there is controversy over the validity of RGC-5 cells. Recent publications emerged claiming that the RGC-5 cell line wasn't derived from rat but from mouse and was actually a photoreceptor cell line 661W. The antibodies against Thy-1 (HIS51) and PAC1 (K-20) used in our studies detect antigens of both mouse and rat origin, so we cannot exclude the possibility that the RGC-5 cell line is from mouse and not rat origin. However, we tend to be reticent about the opinion that RGC-5 cells are ganglion cell-like cells, because the RGC-5 cells used in our studies were positive for Thy-1 labeling, which is a RGC specific marker. In our experience, similar to RGCs, the RGC-5 cells used were sensitive to oxidative stress and neurtrophin withdrawal. 661W cells resemble neuronal cells, demonstrating cellular and biochemical characteristics exhibited by cone photoreceptor cells [25]. The authors deduced that the 661W cell line was also present in the RGC-5 originating laboratory, which probably resulted in cross contamination. If this is the reason, then we cannot help but wonder how the authors ensure that their conclusion wasn't also a mischaracterization due to cross contamination since the phenotypic origin of RGC-5 was detected with 661W? Also the hypothesis of RGC-5 cells being photoreceptors cannot explain the evident labeling for Thy-1 in the current studies. As the two cell types are very different by molecular phenotyping, it is difficult to reconcile this discrepancy. In fact, the same group who published this recent report previously used RGC-5 cells for other studies, confirming their retinal ganglion cell origin [26]. Even though RGC-5 cells are not so ideal a model of RGCs as they were once considered, they still represent neuronal precursor cells which are useful for future neuroprotection type of investigations.

The effects of PACAP on apoptosis have been studied in numerous *in vitro* and *in vivo* models. PACAP influences apoptotic signaling at various levels from initiation to downstream cytosolic and mitochondrial pathways and finally affecting executor caspases [4]. Previous studies suggest that the protective effects of PACAP involve several mechanisms. Different apoptosis-related markers including p-Akt, extracellular signal-regulated kinase, PKC, Bcl-2, p-p38MAPK and activated caspases (8, 3, 12) [27], JNK, apoptosis inducing factor, cytochrome C, phospho-Bad [28] and p53 [29] have been investigated to demonstrate that inhibition of apoptosis is one of the PACAP-induced pathways with therapeutic potential. In the current studies, UVB-irradiation to RGC-5 cells results in ROS over-generation, with reduction of Bcl-2 and up-regulation of Bax, along with loss of mitochondria membrane potential loss and activation of caspase-3 as revealed in our previous studies [24]. This pathway represents the classic mitochondrial or intrinsic pathway to apoptosis. In agreement with previous studies about PACAP, CHC attenuates the apoptosis of RGC-5 cells by affecting the aforementioned central coordinators of mitochondria-mediated apoptosis pathway.

Oxidative stress plays an important role in the pathophysiology of glaucoma [30]. Because of the central role played by mitochondria as both a source and target of oxidative stress, therapeutic agents targeting mitochondria are proposed to protect RGCs from glaucomatous damage. Considering previous report that demonstrated oxidative stress in RGC-5 cells caused by UVB light [31], we adopted the UVB-irradiation paradigm to determine whether CHC provides protective effects via maintaining mitochondria function. Our study found that exposing RGC-5 cells to a small dose of UVB irradiation could lead to marked reduction in cell viability, which was attenuated by CHC at micro molar concentrations. Hoechst/PI staining also provided evidence for this finding.

Reactive oxygen species are formed in the eyes following a wide variety of stressors, and are largely implicated in glaucoma pathogenesis [32].

Mitochondria are the major source of superoxide production and are subject to the direct attack by ROS [33].Oxidative stress occurs when cellular production of ROS exceeds the capacity of anti-oxidant defenses. As proved by previous studies, mitochondria dysfunction could lead to increased production of ROS which could aggravate oxidative stress [34]. Neuroprotective candidates like sulbutiamine [35], isoquercitrin [36] and crocetin [37] have been demonstrated to exert ROS inhibitory effects on RGC-5 cells, but no study has investigated the ability of PACAP analogs to scavenge ROS in RGC-5 cells. Our study demonstrated for the first time that the increase in ROS due to UVB exposure was significantly reduced by the pretreatment of 10 µM CHC, indicating the inhibitory effect of CHC on mitochondria dysfunction and oxidative stress.

Additionally, the expression of Bcl-2 in UVB-exposed cells was decreased significantly compared to that of normal control cells (P<0.01). Bcl-2 family members like Bax promote apoptosis, whereas other members including Bcl-2 and Bcl-Xl exert anti-apoptotic effects [38]. In UVB+CHC group, Bcl-2 was up-regulated accompanied with the down-regulation of Bax. The execution of apoptosis is usually associated with an up-regulation of Bax, which is a pro-apoptotic marker. The increased levels of Bax in the cell then combine with the Bcl-2 already present in the cell. This lowers the amount of free Bcl-2 in the cell, driving the cell toward apoptosis [39]. Bax and Bcl-2 proteins closely interact with the component forming the mitochondria permeability transition pore that allows proteins to escape from mitochondria into cytosol to initiate apoptosis [40]. In the current study, UVB irradiation resulted in ROS accumulation, an increase in Bax and a decrease in Bcl-2 besides mitochondrial membrane potential loss and caspase-3 activation as revealed by our previous findings [24].

Excessive activity of NMDA-type glutamatergic channels has been implicated as a mechanism for neuronal injury in neurologic disorders, including glaucoma [41]. Previous studies have demonstrated that intravitreal injection of NMDA resulted in significantly increased number of apoptotic RGCs and exogenous PACAP is able to counteract NMDA-induced toxicity [42]. Since neural retinal degeneration induced by NMDA has been well established as an experimental glaucoma model, we adopted this model to investigate the protective effects of CHC.

ERG has been considered as a more sensitive method to illustrate retinal injury than histology [43]. The amplitude of PhNR is well recognized as a marker to reflect the function of RGCs [44]. The b-wave mainly originates from bipolar cells that are post synaptic to photoreceptors [45]. Reduced amplitudes in b-wave and OPs are commonly seen in the early stage of retinopathy even before its onset [46]. With CHC administration, the amplitude of PhNR was increased from 1/3 to approximately 2/3 of the controls compared to eyes treated with NMDA only, suggesting marked recovery of RGC function. The functional outcomes, though not absolutely parallel with the morphological observation, was significantly improved after CHC treatment, indicating that, similar to PACAP [47], the protective effects of CHC could transfer to functional improvement.

Our morphometric analysis showed that intravitreal injection of CHC at different concentrations results in less cell loss in GCL. It was reported that the neuroprotection conferred by PACAP in NMDA-induced mice retinal damage was optimal at 100 pM increasing GCL cell number by about 16% of control, while no bimodal response was observed [42]. In our studies, the protective effect of CHC on the retina is not strongly dose-dependent, with optimal effects acquired at 10 µM and 10 pM respectively, increasing GCL cell number by around 20% of control. Similarly, it has been reported that in transient ischemia model induced by high ocular pressure in rats, the neuroprotective effect of PACAP is bimodal with a concentration peak of 10 fM related to the MAP kinase cascade and another peak of 10 to 100 pM reflecting the cAMP cascade [48]. The neuroprotective effect of PACAP in neuronal/astroglial cultures is also bimodal, with peak effects observed at a subpicomolar and a nanomolar concentration [49]. Emerging evidence suggests that nanomolar concentrations of PACAP affect neurons directly, while subpicomolar concentrations exert an indirect glial-cell-mediated protective effect [50]. Our results regarding the bimodal effect of CHC in the retina may suggest the involvement of two different pathways. Due to the difference in animal type and NMDA dosage, the results in the current studies aren't quite comparable to previous ones where NMDA amount ranged from 5 nmol to 200 nmol [51], [52] and animal type varied between C57BL/6 mice and SD rats [53]. Besides, as is stated above, the results of previous studies aren't consistent either. The comparison of the protective mechanism and efficiency between the two different peptides will be further investigated in our future *in vivo* studies.

In conclusion, the present study demonstrates the neuroprotective effect of C*HSDGIC* from the cyclization of PACAP (1–5) against RGC apoptosis in both *in vitro* and *in vivo* models. CHC exerts protective effects through the mitochondria pathway. Analogs of PACAP, especially selective PAC1 agonists like CHC may have a therapeutic value in glaucoma as an adjuvant treatment modality.
